# Daily stress as link between disadvantage and smoking: an ecological momentary assessment study

**DOI:** 10.1186/s12889-019-7631-2

**Published:** 2019-10-12

**Authors:** Tina Jahnel, Stuart G. Ferguson, Saul Shiffman, Benjamin Schüz

**Affiliations:** 1 College of Health & Medicine, University of Tasmania, Hobart, Australia; 2 Department of Psychology, University of Pittsburgh, Pittsburgh, USA; 3 Institute for Public Health and Nursing Science, University of Bremen, Bremen, Germany

**Keywords:** Social disadvantage, Smoking, EMA, Daily stress

## Abstract

**Background:**

There is a well-established social gradient in smoking, but little is known about the underlying behavioral mechanisms. Here, we take a social-ecological perspective by examining daily stress experience as a process linking social disadvantage to smoking behavior.

**Method:**

A sample of 194 daily smokers, who were not attempting to quit, recorded their smoking and information about situational and contextual factors for three weeks using an electronic diary. We tested whether socioeconomic disadvantage (indicated by educational attainment, income and race) exerts indirect effects on smoking (cigarettes per day) via daily stress. Stress experience was assessed at the end of each day using Ecological Momentary Assessment methods. Data were analyzed using random effects regression with a lower-level (2-1-1) mediation model.

**Results:**

On the within-person level lower educated and African American smokers reported significantly more daily stress across the monitoring period, which in turn was associated with more smoking. This resulted in a small significant indirect effect of daily stress experience on social disadvantage and smoking when using education and race as indicator for social disadvantage. No such effects were found when for income as indicator for social disadvantage.

**Conclusion:**

These findings highlight the potential for future studies investigating behavioral mechanisms underlying smoking disparities. Such information would aid in the development and improvement of interventions to reduce social inequality in smoking rates and smoking rates in general.

## Background

It is well documented that there is a substantial social gradient in smoking behavior: among smokers, those who are more socially disadvantaged also smoke more heavily [1–3] and suffer worse health implications as a result [4, 5]. In addition, the knowledge of, access to, and use of healthcare resources vary widely across population subgroups [6]. The concentration of smoking among socially disadvantaged groups is based on complex individual and social processes and are fundamental to understanding the persistent unequal distribution of smoking [7]. However, to date, little is known about the processes through which social disadvantage translates into smoking behavior.

Harwood and colleagues [8] posited that psychosocial factors, such as perceived stress mediate the relationship between socioeconomic status (SES) and smoking. The unequal distribution of socioeconomic resources and the resulting socioeconomic disadvantages imply greater stress experience for those with less access to resources [9, 10]. In addition, people who experience socioeconomic disadvantage also experience more stressful events and have fewer resources to successfully cope with this stress [10–13]. This, in turn, might lead to increased smoking as a maladaptive way of coping with the stress [14]; essentially, that some people may engage in smoking as a means of coping with stress [15, 16].

Providing enhanced cessation support to smokers experiencing stress [17, 18] could therefore help to reduce smoking prevalence, particularly among socially disadvantaged smokers [10]. This idea implies a mediational relationship: Smoking is linked indirectly (mediation) to socioeconomic disadvantage through the experience of everyday stress, i.e., people would be assumed to smoke more as a response to experiencing higher levels of stress in their everyday lives through socioeconomic disadvantage. While this assumption is both plausible and supported by research on either ends (disadvantage leads to higher levels of daily stress [19]; and higher levels of daily stress are associated with higher levels of smoking [20], relatively few studies have investigated the role of daily stress in the social gradient in smoking.

Most previous studies examining the role of stress on the social gradient in smoking have operationalized stress as major live events or chronic stress [12, 21]. For example, Mulder and colleagues reported that multiple chronic stressors (e.g., financial problems, low perceived life control and lack social support) were found to be directly related to smoking and partially mediated the relationship between educational level and smoking behavior [10]. In essence: Socially disadvantaged individuals experience more stressful situations which is associated with higher smoking rates, compared to less disadvantaged individuals [10, 13].

This view, however, is based on the idea that there are differences between individuals in how much chronic or acute stress they experience, and that these differences translate into stable differences in smoking rates between individuals (between-subject levels of analysis [22, 23];). Such analyses cannot explore whether smokers smoke more cigarettes in situations or on days when they are more stressed (within-subject level of analysis [24];). Where studies using between-subject level of analysis aim to compare groups of people, ascertaining whether situations in which individuals are stressed cues smoking calls for within-subject level of analysis. A previous study examining the link between the experience of daily stress and smoking for example showed that individuals reported smoking significantly more in situations when they experienced more stress [20]. In addition, daily stress captures day-to-day experiences that potentially offer insight into the circumstances that may facilitate or preserve social inequalities in health [19] and therefore might capture some of the variance in the association between aspects of social disadvantage and smoking. Grzywacz et al. [19], for example, found that exposure to daily stress was status related: severity of stressful situations for lower SES individuals was higher than for individuals with higher SES. However, the potential of daily stress experience as a factor linking socioeconomic disadvantage and smoking has not been formally tested yet.

Examining the role of daily stress on the social gradient in smoking has both theoretical implications and may guide and inform the design of interventions targeted in particular at smokers with low socioeconomic status through reducing individual stress experience. The goals of these secondary analyses are to investigate whether social disadvantage, assessed through the unequal distribution of socioeconomic indicators, induces different levels of daily stress smokers experience and whether this is associated with smoking. It is hypothesized that perceived daily stress indirectly links indicators of social disadvantage and smoking in a way that more socially disadvantaged smokers perceive more daily stress, and that greater stress perception is in turn associated with more cigarettes smoked.

## Methods

### Overview

To explore our research question, we conducted secondary analyses using data from a larger real-world study looking at smoking patterns [25, 26]. The study used Ecological Momentary Assessment (EMA) methods to assess smoker’s daily stress experiences and smoking as they went about their day-to-day lives. This data collection strategy allows us to examine fluctuations in daily stress and smoking, while taking individual differences (e.g. education and income) into account. The study was approved by the Institutional Review Board at the University of Pittsburgh (REN16120111 / IRB0606147).

### Participants

Details on the recruitment methods have been reported elsewhere [25, 26]. Briefly, between November 2007 and January 2010, 194 daily smokers were recruited in the Pittsburgh area. The study was introduced to the participants as a naturalistic study of smoking patterns. Eligibility criteria included being a smoker for at least three years, report smoking every day (defined as smoking 5 to 30 cigarettes per day [CPD]) for at least the last 3 months, not planning to quit within the next month, and be at least 21 years old. The presence of diagnosed mental health conditions were not listed in the exclusion criteria. Written informed consent was obtained, using a consent form approved by the University of Pittsburgh Institutional Review Board (IRB).

### Procedure

After reviewing and signing a consent form, participants completed a baseline questionnaire including information about basic demographic characteristics (age, gender, educational attainment, income, racial background). The EMA monitoring procedures have been reported previously [25, 26]. Data collection took place using hand-held electronic diaries (EDs). Participants were supplied with an ED running data collection software programmed specifically for the study. Participants received hands-on individual training to monitor their smoking, activities, feelings and social setting in real-time. Participants were asked to carry the ED at all times during the waking day. Every night, participants were also asked to complete an evening report and state the severity of their perceived stress on any given day. Additionally, participants were given the opportunity to use the evening report to record extra cigarettes they had missed to report in real-time. Participants monitored their smoking over 21 days (M = 22.5, SD = 4.12).

### Measures

To identify those who are most and least socially disadvantaged, individuals were classified according to their educational attainment, income, and racial background (all indicators were assessed during the baseline visit). The statuses people occupy as adults and the resulting social disadvantages/ advantages can be the consequence of many factors. Although these factors are correlated with one another, they are only partially overlapping and exert their effects on behavior differentially and therefore can provide opportunities or barriers towards behavior [27]. Hence, we need to examine different socioeconomic indicators to determine their association with the experience of stress and smoking.

Higher education, for example, indicates easier access to and better processing capability of health-related information [28]. Education is usually established early in life, is generally stable throughout the life course and is in particular important as it is associated with future economic resources and knowledge about health-related strategies (e.g. strategies to cope with stress [29];). Moreover, educational attainment has been a widely used proxy for social disadvantage in previous studies and it is less prone to endogeneity bias from reverse causality (smoking effecting educational attainment) than measure such as income. Those with lower educational attainment, therefore, are considered as more socially disadvantaged. Education was dichotomized as lower education (“8^th^ grade or less”, some high school, no graduation/ GED”, “high school graduate/ GED”; coded as higher social disadvantage = 1) and higher education (“some college”, “college graduate/ degree”, “some graduate work”, “graduate degree”; coded as lower social disadvantage = 0).

Income, in contrast, captures the resources individuals can access to purchase goods and support in times of need and defines the possibility someone has to afford prestige in society [11]. For example, individuals with low income may lack the ability to purchase goods and services that reduce stress or minimize the sources of stress [14]. Lower income therefore can also represent a proxy for social disadvantage. Income was operationalized as the annual household income in US dollar. To ensure balanced groups sizes, income was dichotomized in low (≤ $14,999; coded as higher social disadvantage = 1) and high income (≥ $15,000; coded as lower social disadvantage = 0).

Finally, we also included racial background as another indicator for social disadvantage. Racial background was dichotomized in “African American” (AA; coded as higher social disadvantage = 1) and “Other” (“Caucasian” and “other”) as most previous studies that address the relationship between stress and smoking in various racial groups have been conducted among African Americans [30, 31]. In particular in the US, individuals with African American background experience substantially more everyday discrimination, which in turn results in higher stress levels, both subjectively experienced as well as verified by biomarkers [32].

The *primary outcome*—CPD—was operationalized by daily cigarette counts assessed through ED logs and daily retrospective reports assessed at the end of the day [33].

The variable assumed to mediate between social disadvantage and smoking (daily stress) was operationalized as the perceived daily stress and was reported by the participant during the evening reports (“Since last Evening Report: Felt nervous/stressed?” with possible answers ranging from 0 to 100). This item has reasonable face validity as it asks directly about stress perception.

### Analysis

**Fig. 1:**
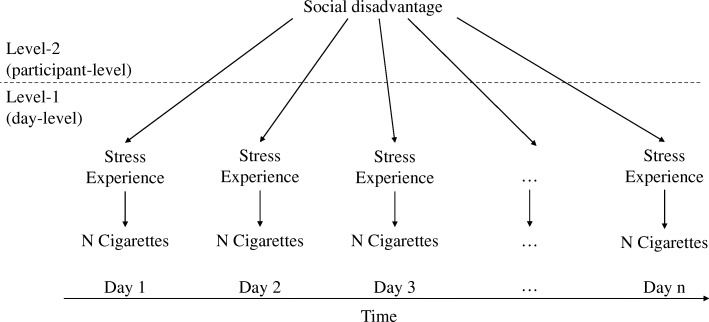
Conceptual diagram of the 2–1-1 mediation model

The aim of this project was the examination of indirect effects of social disadvantage indicated by education, income and racial background (independent variable) on smoking (dependent variable) via perceived daily stress (mediator [34];). Because of the hierarchical structure of EMA data with multiple assessments nested under participants, the non-independence of observations was taken into account using multilevel analysis. For the analysis, 2–1-1 mediation models with random intercepts and fixed slopes were used [35]. This means that the independent variable (social disadvantage) was measured on the between participant level, whereas the dependent variable (CPD) and the mediator variable (perceived daily stress) were assessed on the within participant level, i.e. via repeated measurement occasions (Fig. 1). The intercepts of the dependent variable (CPD) and of the mediator variable (perceived daily stress) were allowed to vary on the between participant level, and their residual variances to correlate. In the analysis we controlled for gender, age and day in study. To estimate these models, MPlus 7 was used (TYPE = TWOLEVEL RANDOM [36];). All analyses controlled for day in the study, baseline CPD and gender.

## Results

Information on participant characteristics in regards to educational attainment, income and race are shown in Table 1. Lower educated participants were significantly older compared to higher educated participants but did not differ in regards to gender or baseline CPD. Participants who reported higher income did not differ in regards to demographic characteristics when compared with participants with lower income. African American smokers were significantly older and reported smoking fewer cigarettes at baseline compared to smokers with different ethnic background.

**Table 1:** Participant Characteristics

	Overall (*n* = 194)	Low education (*n* = 81)	High education (*n* = 113)	Low income (*n* = 91)	High income (*n* = 103)	African American (*n* = 73)	Other Ethnicity (*n* = 121)
Age	41.2 (11.2)	42.6 (10.6)**	40.0 (11.5)	40.9 (11.2)	41.5 (11.2)	43.6* (9.8)	39.7 (11.7)
Gender (Male)	55.2% (*n* = 107)	56.8% (*n* = 46)	54.0% (*n* = 61)	54.9% (*n* = 50)	55.3 (*n* = 57)	46.6% (*n* = 34)	60.3% (n = 73)
CPD	16.1 (6.5)***	16.5 (6.4)	15.7 (6.6)	15.9 (7.0)	16.2 (6.0)	14.2** (6.0)	17.2 (6.6)

Entries are *M* (*SD*), unless % is specified. *M* = mean, *SD* = standard deviation, *CPD* = Cigarettes per day. Comparisons were made using t-tests (continuous variables) or chi-square tests (categorical variables). * *p* < .05, ** *p* < .01, *** *p* < .001

The results for the multilevel mediation analysis are shown in Table 2. Participants with lower educational attainment perceived significantly more daily stress than participants with higher education. On days during which participants perceived more daily stress a higher number of cigarettes were smoked. This resulted in a small significant indirect effect of education via perceived daily stress on CPD while controlling for the direct effect of education on CPD, baseline CPD and gender. The findings show that perceived daily stress partially mediates the effect of education on CPD.

**Table 2:** Coefficient Estimates for parameters in 2–1-1 mediation model predicting CPD with education, income and race as indicators for social disadvantage

Coefficient Estimates	Education	Race	Income
Fixed Effects (Measurement occasion level)			
Intercept CPD	1.99 (1.13)	2.20 (1.10)*	1.74 (1.13)
Intercept Daily Stress (DS)	71.52 (2.56)***	76.00 (1.41)***	78.98 (1.45)***
Social Disadvantage (D) → DS	8.42 (2.81)**	6.95 (2.10)***	−0.82 (2.18)
DS → CPD	0.01 (0.01)**	0.01 (0.01)**	0.01 (0.01)**
Direct Effect SD → CPD	−0.53 (0.66)	−1.39 (0.55)*	−0.35 (0.61)
Indirect Effect	0.12 (0.04)*	0.10 (0.05)*	−0.01 (0.03)
Total Effect	−0.42 (0.65)	−1.29 (0.54)*	−0.37 (0.61)
Random Effects (Participant level)			
Residual Variance Intercept DS	203.07 (19.18)***	202.08 (18.92)***	212.89 (19.21)***
Residual Variance Intercept CPD	16.16 (1.97)***	15.79 (1.91)***	16.17 (1.96)***
Covariance CPD DS	12.61 (0.81)***	12.61 (0.81)***	12.61 (0.81)***

Entries are *B (SD), B* = coefficient estimates, *SD* = standard deviation, *CPD* = Cigarettes per day, *DS* = Daily stress, *D* = Social Disadvantage. *p < .05, **p < .01, ***p < .001

When using race as indicator for social disadvantage, the results show that AA smokers reported smoking fewer cigarettes than non-African American smokers*.* However, AA smokers perceived significantly greater daily stress averaged across days of the monitoring period compared to non-AA individuals. Higher levels of average perceived stress within individuals in turn was related to greater increases in CPD when controlling for baseline CPD. This resulted in a significant indirect effect of racial background via perceived daily stress on CPD while controlling for the between-person direct effect of racial background on CPD. This indirect effect is the result of higher intercepts of daily stress and less CPD for AA smokers compared to non-AA smokers (Table 3). When separating the sample by race, the results show a non-significant negative effect of daily stress on CPD among AA smokers and a non-significant positive effect of daily stress on CPD among non-AA smokers. No indirect or direct effects were found for income as indicator of social disadvantage.

**Table 3:** Estimates for daily stress and CPD for AA and non-AA smokers

	AA	Non-AA
Intercept CPD (*SD*)	9.952 (1.630)	10.263 (1.213)
Intercept Daily Stress (*SD*)	82.542 (1.633)	76.00 (1.41)
Daily stress → CPD *B* (*SD*)	−0.013 (0.018)	0.012 (0.015)

*SD* = standard deviation, *CPD* = Cigarettes per day

## Discussion

Using a within-subjects design, the aim of this project was to examine the role of the experience of daily stress as an intermediary process linking social disadvantage to cigarette consumption in an US sample of smokers. The results suggest that those individuals who are socially disadvantaged in terms of education and racial background experienced more daily stress. Those who experienced more daily stress in turn smoked more cigarettes, resulting in significant indirect effects of education and racial background on smoking, mediated by stress. Although the within-person positive indirect effect indicates that AA smokers are more affected by daily stress experience, the direct effect showed that AA participants smoked less overall compared to smokers from other racial backgrounds. This is in line with previous research suggesting that differences in CPD for AA and non-AA smokers may be explained through biological markers as AA smokers have a slower rate of nicotine metabolism [37] and that smokers who have a faster rate of nicotine metabolism smoke more cigarettes per day [38].

The findings suggest that the experience of daily stress might function as one possible factor linking social disadvantage and smoking [14, 21]. Further, the results support previous findings that social disadvantage is associated with perceived stress [39]. In addition, it could be shown that the association between social disadvantage and the experience of daily stress differed according to the indicators of social disadvantage used in this study. This suggests that the mechanisms of how social disadvantage translates into daily stress experience differ, depending on which indicator for social disadvantage is used. Each indicator of social disadvantage highlights important differences in terms of scope and implications on smoking, and although they overlap in how they affect smoking behavior, they should not be used interchangeably. We can only speculate, but for example, individuals with lower educational attainment may be more vulnerable to daily stress because their stressors are more severe and are experienced as more disruptive to their daily lives through a lack of material and psychological coping skills [40]. Individuals with lower income may experience rather long-term stress in the form of financial strain, which may not necessarily translate into daily stress experience, but rather chronic stress.

Some limitations need to be noted when interpreting the findings. Firstly, we used a crude single-item measure of daily stress experience which may not adequately reflect how much stress participants experienced on a day-to-day basis. However, other studies have found that single-item stress measures are both reliable and valid, perform comparably to longer stress scales [41] and have been successfully tested in real-world studies [42]. Future research on the association between daily stress and smoking may consider collecting data on stress experience not just at the end of each day, but at multiple time points per day in order to get a more accurate measure of the intensity of daily stress. Assessing the different types of stressors linked to the stress experience may also yield deeper inside into the effects of stress on smoking. In addition, for social disadvantage, we used proxy measures such as education, income and racial background. Other indicators such as occupation, that tap explicitly into a person’s standing within a society may yield further inside into the effects of social disadvantage and smoking. Nevertheless, the indicators used in this study have been widely used in health research [43]. Future studies might consider more comprehensive measures of social disadvantage in order to better capture differences in stress and smoking related to socioeconomic status. In order to gain sufficient and comparable sample sizes we dichotomized the indicators which may have minimized their effects on daily stress experience and smoking. Larger samples would yield the opportunity to explore effects of social disadvantage on daily stress experience and smoking more decidedly. For an EMA study however, the sample size was relatively large considering the vast amount of data collected for each individual participant.

Additionally, we have explored the effects of social disadvantage and daily stress experience on CPD, as the most important driver of nicotine intake. Other factors, such as puffing topography, or nicotine extraction per cigarette might also be important indicators of smoking as a means to relieving stress. In the context of an EMA study however, these factors would be difficult to measure and potentially add substantial participant burden. However, further research may explore ways to passively detect smoking instances, including puffing topography and how they relate to social disadvantage and daily stress experience.

Further, EMA data is correlational in nature which make causal interpretations difficult. Thus, reverse causation explanations (i.e. smoking causing stress) are also plausible. Some models such as the stress induction model posits that smoking induces stress [44]. It has also been suggested that smoking may induce (financial) stress and that stress may lead to smoking induced deprivation [45]. However, the findings of Study 1 are supported by experimental studies, showing that the experience of stress reduces the ability to resist smoking and increases both smoking intensity and reward [46, 47]. Further, self-reports and situational characteristics have the potential to induce reactivity, which means that the monitoring itself might change participant’s behavior. To date, research on the potential that EMA methods might have on reactivity are mixed [48, 49]. Nevertheless, EMA methods represent a substantial improvement over more common retrospective methods, as they maximize ecological validity, while avoiding recall bias [50].

It must also be noted that the significant effects we found were only small (see Table 2). Hence, perceived stress does not offer a comprehensive explanation of how educational attainment and racial background might translate into smoking behavior. Other variables, such as the environment, social norms and networks may represent other processes linking SES and smoking [51]. However, as we conducted secondary analysis for this study, socioeconomic variables were not the main focus when data was originally collected.

Lastly, the data collection ended about 9 years ago. A substantial amount of time has passed, in which smoking related public health interventions, changes in the costs of living etc. may have had significant effects on the relationship between social disadvantage and smoking. With rising inequalities however, we would expect the effects of social disadvantage on daily stress experience and smoking to be even stronger today. Replication of this study with more recent data is needed to confirm the results from our study.

In summary, the findings highlight the need for more focused research, with more diverse samples and better developed theory, in order to better understand the role of daily stress on social inequalities in smoking behavior. From an intervention perspective, examining the link between stress and smoking is important as smoking is suggested to be a maladaptive coping response to stress [16]. Some social support interventions target stress management and coping skills [52, 53]. As noted, the limited number of studies conducted to date broadly supports the premise that social support interventions targeting better management of daily stress may improve cessation outcomes, especially when focusing on socially disadvantaged smokers. The findings from this work, however, suggest that the direct effect of stress on day-to-day smoking is likely minimal, suggesting that additional work is required to understand how such interventions should be utilized.

## Conclusion

The current study extends previous work on stress related effects on smoking by taking a within-participant and time-varying perspective. Using real-time, ecologically valid assessments of daily stress experience and smoking, we examined whether people experience, as a function of different indicators of social disadvantage, different levels of daily stress, and whether this affected smoking differentially. The mixed findings and small effect sizes highlight the need for future studies investigating whether and how the experience of daily stress influences smoking and smoking cessation. Such information would aid in the development and improvement of social support interventions specifically tailored to socially disadvantaged populations that focus on healthier coping skills and daily stress management. In order to better understand the social gradient in smoking behavior future research may focus on investigating the effects of different indicators of social disadvantage on stress experience and the types of stress (e.g. daily hassles) and how this affect daily smoking.

## Data Availability

The dataset used and analysed during the current study are available from the corresponding author on reasonable request.

## Abbreviations

AA: African American
CPD: Cigarettes smoked per day
DS: Daily stress
ED: Electronic diary
EMA: Ecological Momentary Assessment
SD: Social Disadvantage
SES: Socioeconomic status
